# Exhaustion, Not Low Engagement, Differentiates Work-Related and Non-Work-Related Depression in Korean Wage Workers: A Negative-Control Analysis

**DOI:** 10.3390/healthcare14183113

**Published:** 2026-09-21

**Authors:** Yangwook Kim, Byung-Yoon Yun, Won-Tae Lee, Hee-Joo Park, Ju-Yeon Oh, Jong-Min Lee, Jian Lee, Jin-Ha Yoon

**Affiliations:** 1 Department of Public Health, Graduate School, Yonsei University, Seoul 03722, Republic of Korea; nor5teo@yuhs.ac (Y.K.); hjpark106@yuhs.ac (H.-J.P.); jyoh@yuhs.ac (J.-Y.O.); cly0310@yuhs.ac (J.-M.L.); leejian83@yuhs.ac (J.L.); 2 Department of Preventive Medicine, Yonsei University College of Medicine, Seoul 03722, Republic of Korea; yby3721@yuhs.ac (B.-Y.Y.); wontaelee@yuhs.ac (W.-T.L.); 3 The Institute for Occupational Health, Yonsei University College of Medicine, Seoul 03722, Republic of Korea

**Keywords:** occupational health, depression, burnout, work engagement, work attribution

## Abstract

**Highlights:**

**What are the main findings?**

- Depression that wage workers associated with work was linked to a measurable, work-related exposure. Exhaustion distinguished work-related depression from non-work-related depression, whereas low work engagement was associated with depression regardless of its attribution to work.
- Using non-work-related depression as a negative control, exhaustion showed a graded difference between work-related and non-work-related depression, whereas lower engagement was equally associated with both types.

**What are the implications of the main findings?**

- Monitoring exhaustion may be more useful than monitoring engagement for identifying work-related depression, as exhaustion is more clearly associated with work-related depression.
- Negative control outcome designs can be applied to routinely collected workplace mental health data.

**Abstract:**

**Background/Objectives**: Workplace mental health surveys routinely measure exhaustion and work engagement, but whether either distinguishes work-attributed depression from depression in general remains unclear. We examined this question among Korean wage workers using a negative control outcome design. **Methods**: We analyzed the seventh Korean Working Conditions Survey (2023), a nationally representative sample of wage workers (complete-case N = 25,776; 270 work-related and 271 non-work-related depression cases). Past-year depression was classified by workers’ attribution, with non-work-related depression as the negative control outcome. This control shares the respondent, instrument, and reporting occasion—and thus the principal reporting biases—but is presumed not to be reachable through an occupational pathway. For each exposure, we estimated the specificity contrast and assessed robustness across adjustment models, propensity-score matching, and stratification. **Results**: The exposures showed a double dissociation. Exhaustion was associated with both depression types, but was more strongly with work-related depression (fully adjusted specificity odds ratio, 1.17 per point; 95% confidence interval, 1.01–1.36), increasing monotonically with severity. Engagement was inversely associated with both, but did not distinguish them (0.97; 0.88–1.07). The dissociation persisted across all adjustment and sensitivity models and under propensity-score matching, where exhaustion did not distinguish between non-work-related depression and no depression—the expected negative-control behavior. **Conclusions**: Exhaustion, but not low engagement, distinguished work-attributed from non-work-attributed depression and may serve as a discriminating surveillance indicator. These cross-sectional associations are consistent with occupational specificity, but cannot establish causality, and they rely on single-item self-reported measures in small subgroups. Thus, replication with validated instruments and longitudinal data is required.

## 1. Introduction

Depression is among the most burdensome conditions in the working-age population [1], and the workplace is widely regarded as both a contributor to, and a setting for, addressing it [2]. A large body of literature links adverse working conditions, including high demands, low control, long hours, and job insecurity, to depressive symptoms [3]. Depression among employed adults also carries substantial costs through absenteeism, reduced productivity, and early labor-market exit [2]. Yet a basic measurement question remains unsettled: how can one determine whether depression in a worker is occupational—and therefore potentially modifiable through workplace intervention—rather than arising from non-work-related causes? This distinction matters for occupational surveillance, compensation and prevention policy, and interpretation of workplace surveys that now include mental health measures. Evidence that occupational injury predicts subsequent depression more strongly than non-occupational injury illustrates the value of distinguishing work-related from non-work-related cases [4]. However, an analogous distinction has not been examined for the psychological exposures most commonly measured in such surveys.

Two work-related states figure prominently in this literature: exhaustion and work engagement. Exhaustion is a core dimension of burnout [5] and is widely considered the burnout component most responsive to work stressors. In contrast, work engagement is a positive, fulfilling state characterized by vigor, dedication, and absorption [6] and is typically treated as a protective resource. The Job Demands–Resources model places these states on separate pathways: exhaustion arises through a health-impairment process driven by job demands, whereas engagement develops through a motivational process driven by job resources [7]. This distinction makes them informative as a pair rather than as a single dimension. Both are routinely measured in occupational health surveys and interpreted as indicators of work-related mental health. Moreover, in a large Korean study, exhaustion mediated the association between occupational stress and depressive symptoms approximately five times more strongly than the disengagement dimension did [8].

However, previous research has not examined whether either state distinguishes depression that a worker attributes to work from depression attributed to other causes. Prior studies have quantified how strongly exhaustion and engagement relate to depression [8,9,10], but they have generally treated depression as a single, undivided outcome. A positive association can therefore have two important interpretations: the exposure may track depression that arises from work, or it may track depression regardless of its origin. An indicator that flags depression irrespective of its source is not equivalent to one that flags depression connected to work. Yet the two are indistinguishable when work-related and non-work-related cases are pooled. Separating them and examining whether exhaustion and engagement behave differently toward them is, to our knowledge, a comparison that has not been made and is the focus of the present study.

One reason the question may have been overlooked is measurement. Instruments designed to capture work-related depression incorporate attribution into depressive symptom items [11], such that reporting a symptom also conveys its perceived cause; these two aspects therefore cannot be separated retrospectively. Critics have argued that symptoms, their possible causes, and causal attributions should instead be measured separately and their relations examined [12,13]. The present data allow this approach because the survey records the depressive condition, its attribution to work, and the two work-framed exposures as separate items. Their correspondence can therefore be examined rather than assumed. This approach also relates to a longstanding debate over whether burnout is distinct from depression, with the same evidence supporting different interpretations [14,15,16]. We do not address that debate here.

Separating these measurements is a precondition, not a solution. Even when the items are measured separately, their associations may still result from confounding or from a general tendency among workers to report both distress and difficult working conditions. In addition, because both the exposure items and the outcome are work-framed, shared work-referenced wording could itself contribute to an observed association. A comparison is therefore needed with an outcome that the exposures should not affect through the occupational pathway under study. Accordingly, we use a negative control outcome design [17,18], which classifies self-reported depression according to whether the worker attributed it to work and treats non-work-attributed depression as the negative control. This control shares the respondent, instrument, and reporting occasion with work-attributed depression and is therefore expected to share the same reporting biases, while presumed to remain outside the occupational pathway under study. An exposure acting specifically through that pathway should be associated more strongly with work-attributed depression than with non-work-attributed depression. Conversely, an exposure that is simply a correlate of depression, or an artifact of shared reporting, should be associated with the two outcomes to a similar degree. Because the control outcome is specified in advance, a null result represents a test that the design has to pass rather than an incidental finding [19,20]. The assumptions underlying this approach, and the conditions under which they may fail, are described in Section 2.3 and revisited in the Limitations.

The two pathways make different predictions once depression is divided according to its attribution. If exhaustion is the endpoint of a demand-driven health-impairment process, its association with depression should be strongest when depression is attributed to work. Similarly, the resource-driven pathway would predict a corresponding differential for engagement. However, we did not expect this difference because engagement has two components that overlap with the diminished energy and loss of interest characteristic of depression. Engagement may therefore also be lower among workers with depression regardless of its origin (Supplementary Figure S1). These two tendencies pull in opposite directions, and a differential association for engagement would be expected to disappear if the non-specific relationship with depression predominates [9,10].

Using the seventh Korean Working Conditions Survey (KWCS), a nationally representative survey of the working population, we therefore examined whether exhaustion and work engagement are differentially associated with work-attributed depression. We hypothesized that exhaustion would be more strongly associated with work-related depression than with non-work-related depression, indicating a differential association consistent with occupational specificity. Conversely, engagement was postulated to be similarly associated with both depression types. In terms of the estimand, we hypothesized that the specificity contrast would exceed 1.0 for exhaustion and approximate 1.0 for engagement. Engagement was treated as a substantive prediction rather than as a null hypothesis of convenience—if both exposures behaved similarly, the results would not support our interpretation. By pairing work-framed exposures with the work attribution of depression, this study offers an approach to separating differential from non-specific associations in routinely collected workplace mental health data.

## 2. Materials and Methods

### 2.1. Data Source and Study Population

This cross-sectional study used data from the seventh KWCS, a nationally representative survey of workers aged ≥15 years conducted in 2023 by the Occupational Safety and Health Research Institute [21]. Modeled on the European Working Conditions Survey, the KWCS collects data on working conditions through computer-assisted personal interviewing of a stratified, multistage sample. Because exposures and outcomes are measured at the same time, the study design cannot establish temporal order. Accordingly, the estimates reported subsequently should not be interpreted as causal effects; throughout, the analysis is framed as a comparison of associations (see Section 2.3).

The analysis was restricted to wage workers for three reasons related to the validity of the study design. First, the outcome requires an interpretable referent: attributing depression to “work” presupposes a distinction between work and non-work. For employees, this distinction is defined by the employment relationship, whereas for self-employed workers, enterprise activities, household economics, and personal circumstances may be less clearly separated. Pooling these groups could therefore blur the contrast on which the negative control design depends. Second, the exposure items presuppose an employment context, referring to the working day and the job. Third, employment type is a recognized social determinant of depression, operating through pathways distinct from the exposures studied here [22]. Including heterogeneous employment arrangements could therefore introduce a confounding structure that differs from the one addressed by our covariate set. This restriction limits the scope of our findings, as discussed in the Limitations.

**Figure 1:**
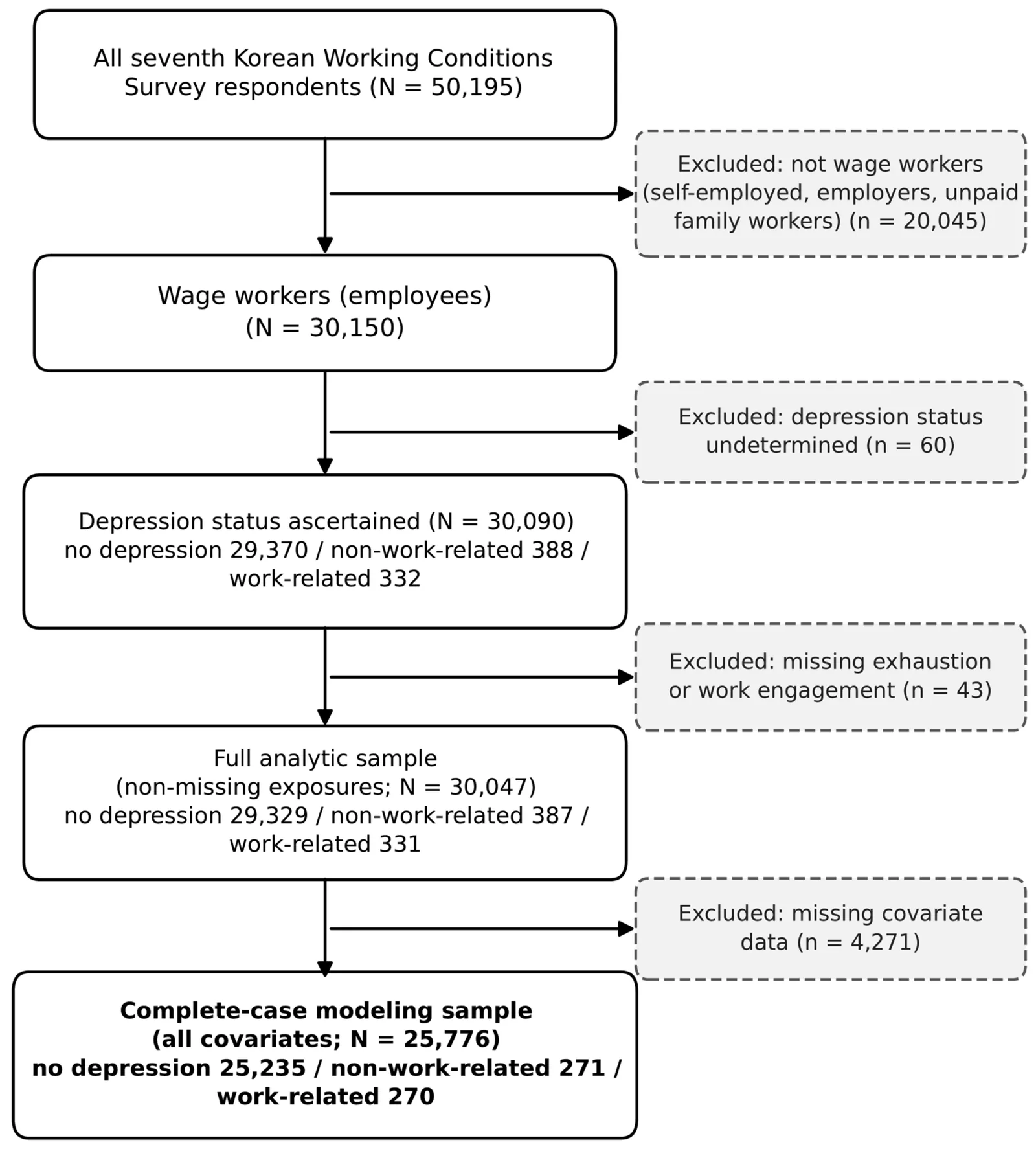
Selection of the study sample from the seventh Korean Working Conditions Survey (2023). Steps 1–4 define the full analytic sample used for prevalence estimates and descriptive statistics, whereas step 5 defines the complete-case sample used uniformly across all regression models. Group counts are shown as no depression/non-work-related depression/work-related depression.

Of the 30,150 wage workers, 30,047 were included in the full analytic sample after excluding participants with undetermined depression status *(n* = 60) or missing exposure data (*n* = 43). Prevalence estimates and population characteristics were reported for this sample. Regression models used a complete-case sample (*N* = 25,776; no depression, 25,235; non-work-related depression, 271; work-related depression, 270), which was applied uniformly to crude and adjusted models for comparability (Figure 1).

### 2.2. Variables

#### 2.2.1. Outcome

Depression was ascertained from the KWCS self-reported health-problems module, in which respondents indicated whether they had experienced any of a list of health conditions over the previous 12 months. Those who reported depression were then asked whether the condition was caused by their work. Based on these two items, we defined three mutually exclusive groups: work-related depression (depression reported and attributed to work), non-work-related depression (depression reported but not attributed to work), and no depression (reference). We use “work-related” and “work-attributed” interchangeably throughout for the first of these groups, and likewise “non-work-related” and “non-work-attributed” for the second. “Work-attributed” more precisely reflects what was measured: the item explicitly asks whether the health problem was “due to your job”—a causal judgement made by the respondent—rather than an independently established work-relatedness. This measure therefore reflects a self-reported depressive condition together with the respondent’s attribution of its cause to work, rather than a clinical diagnosis or validated symptom scale. The non-work-related group serves as the negative control outcome (see Section 2.3).

#### 2.2.2. Exposures

Exhaustion and work engagement were measured using five items from the KWCS work-engagement module, each rated on a five-point frequency scale and recoded so that higher scores indicated greater frequency. The three engagement items comprise the Work Engagement Scale-3, whose reliability and validity have been assessed among Korean workers using KWCS data [23]; the two exhaustion items have not been validated in the same manner. Engagement was calculated as the sum of three items assessing whether participants felt full of energy at work, enthusiastic about the job, and that time passed quickly while working (range 0–12). Exhaustion was the sum of two items assessing physical exhaustion at the end of the working day and emotional drain from work (range 0–8). Thus, the two subscales differed in both length and validation status.

Internal consistency in the present sample was adequate for both subscales and did not favor the longer one. Cronbach’s alpha was 0.80 for the three engagement items, while the two exhaustion items correlated at r = 0.78, corresponding to a Spearman–Brown coefficient of 0.88 (Supplementary Table S6). Coefficients were near-identical across the three depression groups (engagement alpha 0.80–0.82; exhaustion Spearman–Brown 0.86–0.88). Both exposures were measured using explicitly work-framed items. For readability and to avoid confusion with work-related depression, we refer to them throughout as “exhaustion” and “engagement”.

Each exposure was analyzed primarily as a continuous per-point score and, secondarily, by score category to display the dose–response pattern. Categories were defined as follows: exhaustion, 0–2 (reference), 3–4, 5–6, 7–8; engagement, 0–3 (reference), 4–6, 7–9, 10–12.

#### 2.2.3. Covariates

Covariates were selected a priori based on an assumed causal structure (directed acyclic graph; Supplementary Figure S1) rather than through data-driven procedures, adjusting only for common causes of the exposure and outcome and not for consequences or mediators [24,25]. Four substantive assumptions underlie this structure. First, exhaustion is treated as the exposure and work-attributed depression as the outcome, with non-work-attributed depression serving as the negative control outcome. Second, work engagement is modeled as an exposure but positioned as a descendant of depression because of its conceptual overlap with anhedonia; consequently, the two exposures are not mutually adjusted in the primary models. Third, sociodemographic and employment characteristics are considered common causes of the exposure and outcome and therefore constitute the adjustment set. Fourth, sleep problems occupy an ambiguous position and are excluded from the primary structure. The second assumption is particularly important to the analysis and cannot be verified using cross-sectional data.

The fully adjusted model included age, sex, education, marital status, personal income, household income contribution, income–expenditure balance, shift type, workplace sector, weekly working hours, workplace size, employment status, and occupation. Two available variables were deliberately excluded from the primary adjustment set and instead reported descriptively for different reasons. Sleep problems could not be assigned a defensible position in the causal structure because sleep disturbance could plausibly be caused by exhaustion, contribute to it, or represent a symptom of depression. Adjusting for a mediator or a descendant of the outcome could therefore introduce overadjustment or collider bias [25,26]. Sleep problems were consequently excluded from the primary models and examined in a sensitivity analysis, in which the resulting attenuation was evaluated (Section 3.4). The WHO-5 Well-Being Index (WHO-5) was excluded on different grounds: because it was measured contemporaneously with the outcome and overlapped with it in content, adjusting for it could remove part of the outcome rather than a confounder. Neither exclusion implies that these variables are unimportant; rather, both decisions follow from the requirement that the primary adjustment set contain only common causes. The stepwise adjustment hierarchy is reported in the Supplementary Material.

### 2.3. Statistical Analysis

All analyses incorporated the complex sampling design of KWCS, including strata, primary sampling units, and post-stratification weight, with wage workers treated as a subpopulation and design-based standard errors estimated using Taylor linearization.

The primary estimand was the differential association of each exposure with work-attributed versus non-work-attributed depression. For exhaustion and engagement separately, we estimated odds ratios (ORs) with 95% confidence intervals (CIs) for three contrasts: work-related depression versus no depression; non-work-related depression versus no depression; work-related versus non-work-related depression (the specificity contrast, estimated by restricting the sample to the two depression groups).

Because the data were cross-sectional, the causal direction could not be established directly. We therefore framed the analysis as a negative control outcome design [17]. If the association of exhaustion with work-related depression is merely an artifact of shared affective confounding or common reporting bias, exhaustion should be associated comparably with non-work-related depression (the negative control). A differential association should instead be consistent with an occupationally specific pathway, although it cannot by itself establish one. Thus, the design addresses potential confounding and shared-method bias but cannot resolve the direction of causation in cross-sectional data. Our use of specificity adapts Hill’s classical criterion [27]: rather than requiring exhaustion to be associated with only one outcome, we assessed whether its association differed between work- versus non-work-attributed depression, representing a graded rather than exclusive form of specificity.

Following the second assumption (Section 2.2.3), the two exposures were separately modeled rather than being mutually adjusted, since adjusting for a descendant of the outcome could constitute overadjustment and induce collider bias [25,26]. This temporal assumption cannot be verified using cross-sectional data. Therefore, we included the mutually adjusted model among the sensitivity analyses (Supplementary Table S1) and did not base the conclusion on a single model specification.

All sensitivity analyses were pre-specified, each addressing a specific concern regarding confounding, mediation, or robustness, and all are reported regardless of significance. One additional analysis using a stricter case definition was conducted post hoc and is identified as such wherever it is reported. The pre-specified analyses comprised a stepwise adjustment hierarchy (M1, demographic variables; M2 and M3, personal and household income and income–expenditure balance; M4, shift type; M5, sector and working hours; M6, workplace size and employment status; M7, occupation, representing the fully adjusted model); mutual adjustment of the two exposures; additional adjustment for sleep problems and job autonomy (considered potential mediators or shared correlates) to assess whether the dissociation persisted despite possible overadjustment [26]; additional adjustment for chronic disease, workplace verbal abuse, and night/weekend work; substitution of industry for occupation; and unweighted estimation (Supplementary Tables S1 and S2).

To assess consistency across subgroups, we estimated the specificity contrast within strata of sex and age and tested for effect modification using interaction terms (Supplementary Table S3). These stratified analyses were underpowered by design because the specificity contrast was estimated within the depressed subsample alone (*n* = 541). They were pre-specified as exploratory and used to assess consistency in the direction of associations rather than to provide definitive tests of effect modification.

Finally, as a model-free triangulation, we matched pairs of depression groups 1:1 by propensity score (estimated from all Table 1 covariates) and compared the matched exposure scores using paired tests. Covariate balance was assessed using standardized mean differences (SMDs), with |SMD| < 0.10 indicating good balance and |SMD| < 0.25 indicating acceptable balance [28,29]. Simultaneous 1:1:1 matching of all three groups did not achieve acceptable balance; therefore, the three group pairs were matched separately. The resulting balance is reported in Section 3.4 (Supplementary Tables S4 and S5).

All analyses were performed using R version 4.5.2 and the survey package for design-based estimation [30]. Statistical significance was set at *p* < 0.05.

Generative artificial intelligence (Anthropic Claude) was used to draft and debug R code for data management, survey-weighted model fitting, and figure production, and to check the code for logical errors; all code was reviewed and executed by the authors, and all reported estimates were verified against the underlying data.

## 3. Results

### 3.1. Sample Characteristics

Past-year depression had a survey-weighted prevalence of 2.15% in the full analytic sample (work-related, 1.05%; non-work-related, 1.11%). Table 1 describes the complete-case sample by depression status; two rows capture the main findings. Mean exhaustion increased monotonically across the three groups, from 3.8 among workers without depression to 4.1 among those with non-work-related depression and 4.6 among those with work-related depression. In contrast, mean engagement fell in both depression groups to a near-identical degree (7.5, 6.5 and 6.6 respectively). Thus, the pattern formalized by the regression models was already visible in the descriptive statistics: exhaustion separated the two depression groups from each other, whereas engagement separated both depression groups from the non-depressed group but not from each other.

The covariates in Table 1 show that the two depression groups are not two versions of the same population, and their differences have implications for the design. Both differed from the non-depressed group in the expected directions: they had a higher proportion of women (67.2% and 52.7% versus 45.1%), lower proportion with a university degree or above (23.5% and 29.2% versus 43.6%), higher proportion reporting that income fell short of expenditure (40.6% and 36.8% versus 22.5%), and higher proportion of workers in manual occupations (42.8% and 39.1% versus 32.1%). Marital status, household size, workplace size, and sector did not differ across the three groups.

Where the two depression groups differed from each other (Table 1), the differences were chiefly in the structure of their employment rather than in demography. Workers with non-work-related depression were the oldest of the three groups (51.5 versus 46.0 and 44.5 years), earned the least (238 versus 280 and 296 × 10^4^ Korean won [KRW]), were least often permanently employed (60.3% versus 76.4% and 77.4%), and worked the fewest hours (33.9 versus 41.4 and 39.0 per week). Workers with work-related depression resembled the non-depressed group in age, income, and employment status but worked the longest hours and were most often on shift or rotating schedules (13.0% versus 6.7% and 7.3%). This profile is what the adjustment set and matched comparisons are intended to address, and helps explain why simultaneous matching of all three groups proved infeasible (Section 3.4).

Sleep problems and chronic disease were elevated in both depression groups relative to the non-depressed group, to a broadly similar degree in each group (sleep problems, 4.2 and 4.4 versus 2.0; chronic disease, 17.9% and 13.2% versus 4.1%). WHO-5 well-being showed a different pattern, decreasing monotonically across the three groups (60.6, 47.9, and 43.7) as exhaustion increased. Sleep problems and WHO-5 were excluded from the adjustment set for the reasons provided in Section 2.2. Chronic disease was likewise not adjusted for in the primary models but was included in a sensitivity analysis (Section 3.4). The corresponding exposure distributions are shown in Supplementary Figure S2.

### 3.2. Per-Point Associations

Each one-point increase in exhaustion was associated with higher odds of both work-related and non-work-related depression, but the association was substantially stronger for work-related depression (Table 2). In the fully adjusted model, the per-point OR was 1.33 (95% CI, 1.19–1.49) for work-related versus 1.14 (1.05–1.25) for non-work-related depression, yielding a significant specificity contrast of 1.17 per point (95% CI, 1.01–1.36; *p* = 0.040), similar to the crude estimate (1.23). Expressed per standard deviation of exhaustion (1.7 points, the standard deviation among workers without depression), the adjusted contrast corresponded to an OR of 1.31.

**Figure 2:**
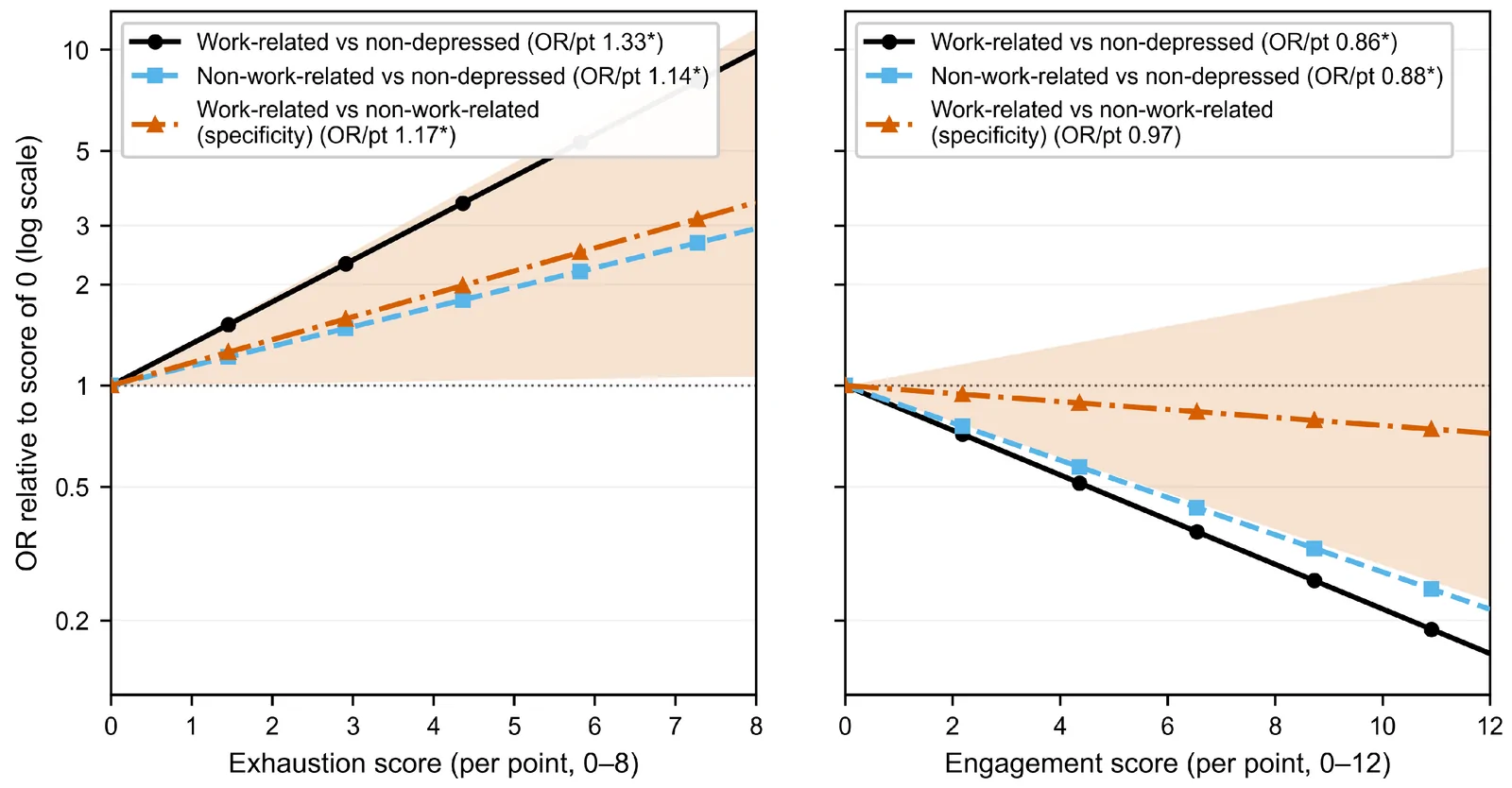
Per-point (continuous) exposure–response curves for exhaustion (**left**) and work engagement (**right**). Each line represents the model-predicted odds ratio relative to a score of 0, from fully adjusted survey-weighted logistic regression, for three contrasts: work-related depression versus no depression (solid), non-work-related depression versus no depression (dashed), and work-related versus non-work-related depression (the specificity contrast; dash-dotted). The shaded band represents the 95% confidence interval for the specificity contrast. An asterisk denotes *p* < 0.05.

In contrast, engagement showed no such differential association. Higher engagement was associated with comparably lower odds of both work-related (0.86; 95% CI, 0.80–0.93) and non-work-related depression (0.88; 0.82–0.95), and the specificity contrast was null (0.97; 95% CI, 0.88–1.07; *p* = 0.57), as in the crude analysis (1.01). These per-point patterns are shown across the full score range in Figure 2.

### 3.3. Dose–Response Across Categories

The differential association of exhaustion with work-attributed depression was dose-dependent (Table 3). The adjusted specificity contrast increased monotonically across exhaustion categories (1.31, 1.84, and 2.77 for categories 3–4, 5–6, and 7–8, respectively, versus the lowest category). This reflects a progressive divergence rather than a null comparison: adjusted odds of work-related depression increased steeply across those categories (2.11, 3.37, and 6.18), whereas odds of non-work-related depression increased only modestly (1.60, 1.79, and 1.95) and were not significantly elevated in the highest category.

**Figure 3:**
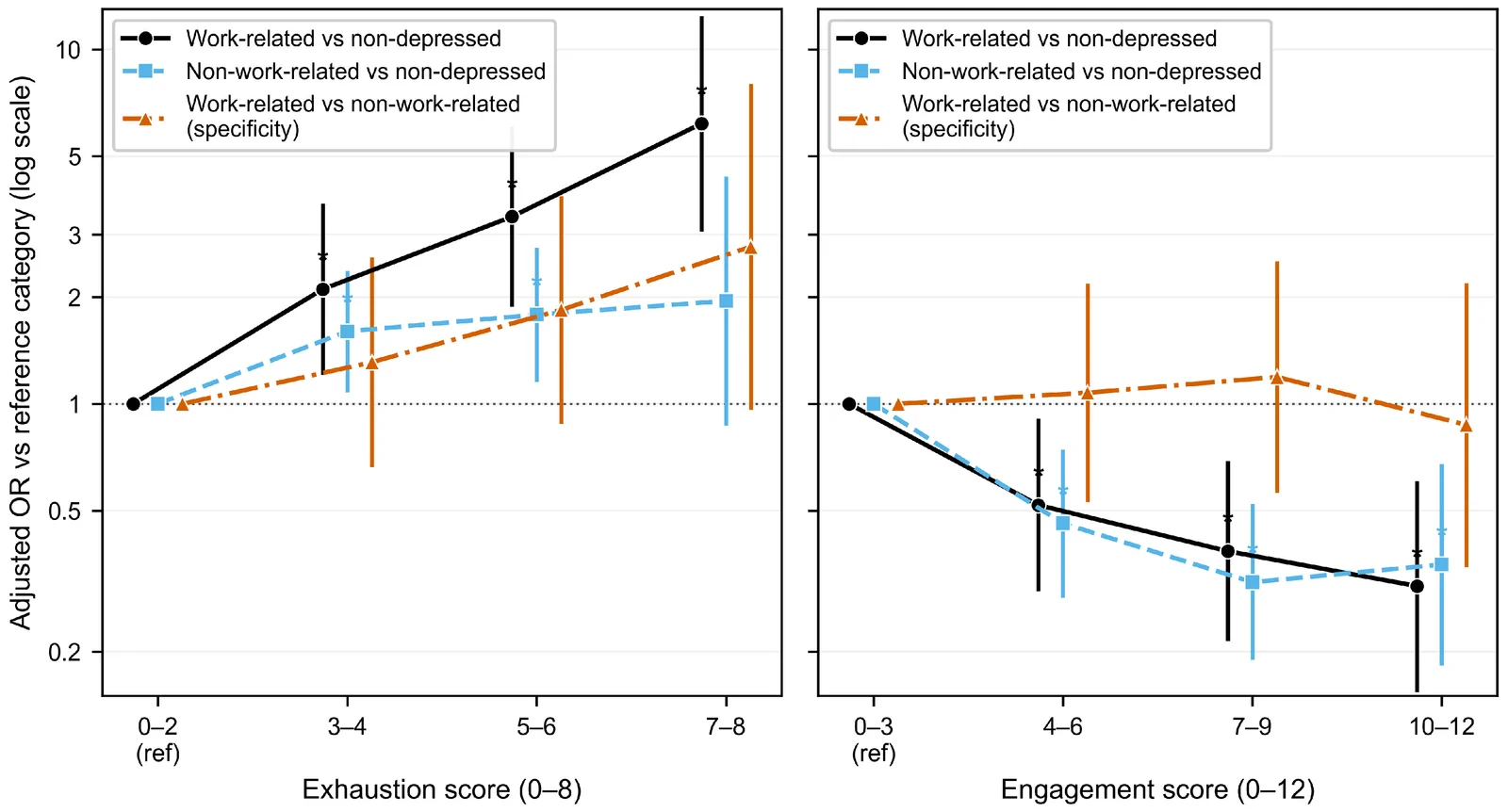
Category-specific (dose-level) adjusted odds ratios for exhaustion (**left**) and work engagement (**right**) from fully adjusted survey-weighted logistic regression. Points represent odds ratios relative to the lowest category (reference); vertical bars represent 95% confidence intervals. The three contrasts are work-related depression versus no depression (solid), non-work-related depression versus no depression (dashed), and the specificity contrast (dash-dotted).

In the crude models, the non-work-related estimates did not reach significance in any category. The precision of the categorical estimates was poor: the highest-category contrast was borderline, with a CI spanning an eightfold range (2.77; 95% CI, 0.97–7.90; *p* = 0.057). Engagement showed no such gradient; although higher engagement categories were associated with lower odds of both depression types, the specificity contrast remained near 1.0 across all categories (adjusted 1.08, 1.19, and 0.87) and never reached significance (Figure 3).

### 3.4. Sensitivity Analyses

Mutual adjustment of the two exposures, which relaxes the temporal assumption underlying our decision to model them separately, did not weaken the contrast (exhaustion, 1.19; 95% CI, 1.02–1.39; engagement, 0.94; 0.86–1.04; Supplementary Table S1). More generally, the exhaustion specificity contrast was consistent across all pre-specified sensitivity analyses, remaining stable in magnitude while varying in statistical significance (Supplementary Tables S1–S5). The continuous exhaustion specificity contrast remained between 1.15 and 1.23 across stepwise and additional regression models and was significant in most. This included adjustment for job autonomy (1.20; 95% CI, 1.03–1.39), chronic disease (1.18; 1.01–1.38), or workplace verbal abuse (1.17; 1.01–1.36); substitution of industry for occupation (1.17; 1.01–1.35); and unweighted estimation (1.18; 1.05–1.33).

The contrast fell just short of significance at three stepwise stages: M2 and M3, which add personal and household income (1.16; 95% CI, 1.00–1.35; *p* = 0.056 and 0.053), and M4, which adds shift type (1.15; 0.99–1.34; *p* = 0.071). The same was observed with additional adjustment for night/weekend work (1.16; 1.00–1.35; *p* = 0.054). It remained significant at M5 through M7 and in the other additional models. Engagement specificity remained non-significant throughout, with estimates ranging 0.94–1.01 (Supplementary Tables S1 and S2).

Additional adjustment for sleep attenuated the exhaustion associations asymmetrically, in the direction predicted by the negative control design (Supplementary Table S2). The non-work-related association fell from 1.14 (95% CI, 1.05–1.25) to 1.06 (0.97–1.16) and became non-significant, whereas the work-related association persisted, decreasing from 1.33 (1.19–1.49) to 1.21 (1.08–1.35). The specificity contrast itself became borderline under this adjustment (1.15; 95% CI, 0.99–1.34).

Stratified analyses showed exhaustion specificity point estimates in the same direction across all subgroups, but the association reached significance only among women (1.30; 95% CI, 1.09–1.55) and workers aged < 45 years (1.33; 1.01–1.75). The estimates were near-null and non-significant among men (1.04; 0.81–1.34) and weaker among those aged ≥ 45 years (1.12; 0.92–1.37). However, neither interaction term was significant, by sex (interaction OR 1.20; *p* = 0.20) or age (0.95; *p* = 0.72). With only 174 men reporting depression, these between-stratum differences in statistical significance cannot be interpreted as evidence of effect modification. We therefore report the stratified estimates as exploratory (Supplementary Table S3; see Section 4).

Propensity-score-matched comparisons, a model-free check, reproduced the dissociation and, more informatively, the behavior expected of the negative control (Supplementary Tables S4 and S5). The negative control comparison behaved as designed: matched non-work-related depression did not differ from no depression on exhaustion (+0.22; 95% CI, −0.07 to 0.50). The comparison between work-related and non-work-related depression showed the expected difference: matched work-related depression had higher exhaustion scores than matched non-work-related depression (+0.36; 0.05–0.67). Engagement showed the expected mirror-image pattern of a non-specific correlate: no difference between the two depression groups (−0.17; −0.63 to 0.28) but lower engagement in non-work-related depression than in no depression (−0.45; −0.84 to −0.07).

Simultaneous 1:1:1 matching of all three groups did not achieve acceptable balance (maximum post-matching standardized mean difference, 0.54), because the non-work-related group differed structurally in age and income. Pairs were therefore matched separately, achieving balance: the maximum standardized mean difference was 0.098 for the work-related versus non-work-related pair, 0.144 for the non-work-related versus no-depression pair, and 0.169 for the work-related versus no-depression pair, with most covariates <0.10 in all three groups (Supplementary Table S5). The comparisons were therefore adequately, though not perfectly, balanced, and their convergence with the model-based results provides additional support for the observed pattern.

Finally, in a post hoc analysis, we repeated the primary models using a stricter case definition that required respondents to report depression and score at or below the standard WHO-5 screening threshold of 50, while retaining work attribution from the original item (Supplementary Table S7). The exhaustion specificity contrast was not weakened but was slightly strengthened (1.26; 95% CI, 1.04–1.52; *p* = 0.020; *n* = 314). The exhaustion association with the negative control outcome, which was significant under the original definition, disappeared completely (1.07; 0.95–1.19; *p* = 0.26), whereas the association with work-attributed depression persisted (1.30; 1.11–1.51). Engagement remained non-specific (0.99; 0.86–1.13).

Two caveats apply. The analysis was post hoc rather than pre-specified, and conditioning on WHO-5 well-being risks collider bias because it is both a consequence of depression and correlated with exhaustion. We would expect such bias to attenuate rather than inflate the contrast; therefore, the finding is better interpreted as an indication that the differential association persists under this conservative restriction, rather than as an improved estimate of its size.

## 4. Discussion

### 4.1. Main Findings

In our nationally representative sample of Korean wage workers, exhaustion and work engagement showed a double dissociation with respect to the work-attribution of depression. Exhaustion was associated with both work-related and non-work-related depression, but substantially more strongly with the former, yielding a significant per-point specificity contrast (OR, 1.17; 95% CI, 1.01–1.36). This contrast was dose-dependent and stable across adjustment and sensitivity analyses, reaching significance in most analyses, although it attenuated to borderline significance in a few. By contrast, work engagement was inversely and comparably associated with both depression types and did not distinguish them (specificity OR, 0.97; Figure 2 and Figure 3). Treating non-work-related depression as a negative control, this asymmetry indicates that exhaustion is differentially associated with the work-attribution of depression rather than depression in general—a pattern consistent with occupational specificity—whereas low engagement is a non-specific correlate. As discussed subsequently, this pattern only weakly constrains interpretation: it does not establish the causal direction for exhaustion and leaves the direction open for engagement (Section 4.3).

The magnitude of these associations also warrants interpretation because a per-point OR of 1.17 is modest in isolation. This specificity contrast compares the association of exhaustion with work-attributed depression against its association with non-work-attributed depression; thus, an OR of 1.17 means that each one-point increase on the 0–8 exhaustion scale is associated with 17% greater odds that a worker’s depression is attributed to work rather than not. Three considerations clarify the practical meaning of this difference. First, one point represents a small unit on an eight-point scale. Expressed per standard deviation (approximately 1.7 points in this sample), the specificity contrast corresponds to an OR of 1.31, while the association of exhaustion with work-attributed depression relative to no depression—the plain association before comparison with non-work-attributed cases—is 1.62 per standard deviation. On the probability scale, survey-weighted predictions from the fully adjusted models show that the prevalence of work-attributed depression increased from 0.30% (95% CI, 0.13–0.47) at the lowest exhaustion score (0) to 2.89% (1.81–3.97) at the highest (8), a 9.6-fold increase and an absolute difference of 2.6 percentage points. Over the same range, predicted prevalence of non-work-attributed depression increased from 0.57% to 1.63%, only a 2.9-fold increase. Engagement showed near-parallel declines for work-attributed (2.87% to 0.47%) and non-work-attributed depression (2.24% to 0.50%). Thus, the dissociation is apparent on the absolute scale as well as in the ORs.

Second, the observed spread between groups exceeded one point: mean exhaustion was 3.8 among workers without depression, 4.1 among those with non-work-attributed depression, and 4.6 among those with work-attributed depression. Across the full scale, categorical estimates also reached 2.77 for the highest versus lowest exhaustion category, although with a wide CI (Table 3). Third, and most importantly for how this finding should be used, our intended application is surveillance rather than intervention. For an indicator, the relevant question is not whether a unit change in the exposure produces a large change in risk, but whether the indicator orders workers in a way that tracks the outcome of interest. A graded association of this magnitude, sustained monotonically across the exposure range, may therefore be informative for surveillance even though it would provide a weak basis for predicting depression in an individual worker. These findings remain cross-sectional associations.

The engagement result also warrants consideration in its own right, rather than serving only as the null arm of the dissociation. Higher engagement was associated with lower odds of both work-attributed (OR, 0.86; 95% CI, 0.80–0.93) and non-work-attributed depression (0.88; 0.82–0.95), with an association comparable in magnitude to that observed for exhaustion. Although engagement did not discriminate between the two outcomes, this does not make the finding uninformative: it is consistent with engagement being associated with depressive symptoms generally, regardless of their attribution. The present analysis therefore indicates that engagement is not suited to the particular task of flagging depression that workers attribute to work, rather than that low engagement is unimportant for worker mental health.

### 4.2. Relation to Prior Work and the Broader Literature

Our central result—a double dissociation in which exhaustion was differentially associated with work-attributed depression, whereas engagement showed no such differential association—relates to two strands of prior research, neither of which has tested this distinction directly.

The first strand concerns the component of burnout more closely linked to depression. In a large Korean sample, exhaustion mediated the association between occupational stress and depression more strongly than disengagement [8]. Similarly, a five-year German cohort provided limited support for the notion that working conditions are more strongly associated with work-related exhaustion than with depressive symptoms [31]. The Job Demands–Resources model offers a rationale for these findings by placing both exhaustion and (dis)engagement on separate pathways: a demand-driven health-impairment process and a resource-driven motivational process [7]. Together, these studies support the view of exhaustion as the burnout dimension most responsive to work stressors [5] and most closely linked to depression. However, they all treat depression as a single outcome and do not examine whether exhaustion is differentially associated with work- versus non-work-attributed depression.

The second strand concerns the work-attribution of depression itself. Because burnout and depression overlap substantially [15], and workers attribute depressive and burnout symptoms to work at similarly low rates (39% and 44%, respectively) [14], exhaustion-centered burnout may not be specifically job-induced. This has motivated instruments that explicitly frame depression as work-caused [11]. Such approaches incorporate or measure attribution but do not test whether it corresponds to a distinct exposure profile. Nor do they address work engagement [6], which we examine alongside exhaustion.

Our analysis brings these two strands together. Using the same work-attribution examined in the burnout literature, we treated non-work-attributed depression as a negative control [17] and asked whether exhaustion is differentially associated with work-attributed depression. In the full analytic sample, 46% of depressed workers attributed their depression to work (unweighted proportion). Exhaustion showed a dose-dependent differential association, whereas low engagement did not. To our knowledge, this is the first study to pair a work-framed occupational exposure with the work-attribution of depression and test their correspondence using a negative control design. Rather than adjudicating whether burnout “is” depression, our findings locate the informative signal in the differential association: depression attribution covaries with a measurable, work-framed exposure in a graded manner.

We interpret this finding no more strongly than the data allow. Both the exposure and the attribution are self-reported, so we cannot exclude the possibility that workers who attribute depression to work are also inclined to report work as draining. However, the negative control indicates that this tendency, if present, does not extend to depression that the same workers do not attribute to work. The finding is therefore consistent with the burnout–depression overlap literature [15], in which exhaustion was also associated with non-work-attributed depression, although more weakly. It also gives a population-based, dose-dependent form to qualitative clinical observations that patients with clinical exhaustion most often attribute their condition to work demands [32].

### 4.3. Interpreting the Dissociation

Several features of the data inform how the differential association of exhaustion should be interpreted. First, the contrast was dose-dependent (Table 3). A graded exposure–response relationship is a long-recognized consideration in causal inference [27] and is difficult to reconcile with a purely artifactual association, although it does not establish direction on its own.

Second, the asymmetry between the two exposures informs interpretation without establishing causality. The differential association of exhaustion is compatible with work demands generating exhaustion that contributes to work-attributed depression or with the reverse ordering; cross-sectional data cannot distinguish between these possibilities. The negative control establishes only that the differential association is unlikely to reflect generic low mood or shared confounding, not that exhaustion causes work-attributed depression.

One asymmetry in our analytic structure also warrants explicit consideration because it could otherwise be mistaken for a finding. The causal diagram positions exhaustion as an exposure and engagement as a descendant of depression. This positioning is an assumption brought to the analysis, grounded in the Job Demands–Resources separation of pathways and the content overlap discussed subsequently. It is not established by these data, and treating the engagement result as confirmation of it would be circular. Two considerations limit the extent to which the argument depends on this assumption. First, the assumption does not affect estimation: both exposures were modeled using the same estimator, covariate set, and contrast. Moreover, the mutually adjusted model, which does not depend on this positioning at all, left the pattern unchanged (exhaustion 1.19, 95% CI 1.02–1.39; engagement 0.94, 0.86–1.04; Supplementary Table S1). Second, the assumption was exposed to potential failure rather than protected from it. Had engagement shown a differential association similar to that of exhaustion, positioning it as a descendant would have been difficult to sustain, and the design was capable of detecting such a pattern. However, it detected no such pattern. We therefore regard the data as consistent with this positioning rather than as evidence for it.

With this qualification, the inference is somewhat clearer for engagement than for exhaustion, although it remains tentative. Engagement was equally reduced in both depression groups. If it were acting as an occupational exposure, it might have been expected to show some preferential association with work-related depression, as exhaustion did. However, the conceptual overlap between engagement and depression is substantial, and its extent determines how much interpretive weight the engagement result can bear. Two of the three engagement components map onto cardinal features of depression: vigor onto the diminished energy of the fatigue criterion and dedication onto the loss of interest that defines anhedonia. Thus, an instrument measuring engagement in a depressed worker partly measures depression itself. In a cross-sectional design, this creates a specific difficulty: an observed association between low engagement and depression is compatible with engagement being a cause, a symptom expressed in the work domain, or a correlate sharing content with depression by construction; the design cannot separate these possibilities. Our finding bears on this question only obliquely: whatever the relationship, it does not depend on the depression being attributed to work.

Reduced engagement may therefore partly reflect a depressed state rather than function purely as an antecedent cause, an overlap also recognized in instruments designed to capture work-attributed depression [11]. On this interpretation, low engagement would function partly as a correlate of depressed state, potentially explaining why it does not show the differential association observed for exhaustion. This interpretation is broadly consistent with longitudinal evidence linking engagement and depressive symptoms over time. A two-wave Norwegian study found significant cross-lagged paths in both directions [10], while a Japanese cohort found that baseline engagement predicted subsequent onset of a major depressive episode [9]. However, this evidence does not specifically support our interpretation. The authors of the Norwegian study judged a normal-causation model superior to a reversed-causation model and concluded that engagement was more likely an antecedent of depressive symptoms than an outcome, while the Japanese cohort examined only the forward direction. Our interpretation therefore rests on the observed reciprocity and the content overlap between the two instruments, rather than evidence that reversed causation predominates. It remains a conjecture that the present design cannot adjudicate.

Third, the dissociation is unlikely to be explained simply by the work-referenced wording of the exposures. Both are equally work-framed: exhaustion items refer to emotional drain from work and physical exhaustion at the end of the working day, whereas engagement items refer to enthusiasm about the job and feeling full of energy at work. Yet only exhaustion showed a differential association. If work-framing alone could produce such an association, engagement should have shown one as well. This feature distinguishes the present design from prior studies that examined either a work-framed exposure or the work-attribution of depression, but not their correspondence.

### 4.4. Robustness and Triangulation

The differential association of exhaustion was robust to analytic choices. Across adjustment steps, attenuation was greatest when working hours were incorporated—a confounder rather than a mediator because working hours precede exhaustion and are themselves associated with depression [33]—yet the specificity contrast persisted after this and other adjustments. Adjustment for sleep problems is particularly informative because it did not attenuate the two associations equally: it eliminated the non-work-related association while the work-related association persisted (Section 3.4; Supplementary Table S2). Sleep disturbance is a plausible shared pathway for exhaustion and low mood of any origin. Thus, an adjustment that removes the negative-control association while leaving the association of interest largely intact argues against the differential being generated by this pathway. The specificity contrast itself became borderline under this adjustment, which is expected when the comparison-group association is reduced toward the null and the subgroup is small. The informative feature is therefore the asymmetric attenuation rather than statistical significance under an admittedly overadjusted model [26].

The same dissociation emerged from categorical dose–response analysis and propensity-score matching. Because these approaches are vulnerable to different biases, their agreement renders an artifact less likely. The categorical estimates should nonetheless be interpreted for shape rather than magnitude. Their imprecision reflects the division of an already small work-attributed subgroup into four levels, with the widest interval spanning nearly an order of magnitude (Table 3); the point estimate should therefore not be interpreted as an estimate of effect size. We treat the continuous specification, which is significant and more precisely estimated, as the primary result and the categorical analysis as evidence that the association is graded rather than threshold-like.

This convergence also addresses group comparability. The two depression groups differed in several characteristics: the non-work-related group was older, worked fewer hours, and had more chronic disease. In a negative control design, the groups need not be identical; rather, the relevant bias pathways must be addressed. The persistence of the specificity contrast after full adjustment and matching that balanced these characteristics (Supplementary Tables S4 and S5) indicates that the differential is not explained by group composition. The matched comparison also reproduced the negative-control behavior directly and provided the most direct evidence that the design worked as intended: matched work-related depression differed from non-work-related depression on exhaustion, whereas matched non-work-related depression did not differ from no depression. This is the pattern expected from a valid negative control—an association with the outcome of interest and not with the control outcome—and it was obtained independently of the regression model.

However, the matched analyses require qualification. Because the non-work-related group differs structurally in age and income, simultaneous matching of all three groups was not achievable. The pairwise matching used in the analysis therefore left residual imbalance that was small but not negligible in the two comparisons involving the non-depressed group (Section 3.4). The matched results should consequently be viewed as triangulation supporting the survey-weighted regression rather than as the primary estimates.

The stratified estimates are exploratory and require cautious interpretation. Although point estimates were in the same direction across sex and age strata, the exhaustion contrast was statistically clear only in women (1.30) and workers aged under 45 (1.33), while it was near-null in men (1.04). Two interpretations are possible, and the present data cannot distinguish between them. The first is statistical: the male depression stratum was small (*n* = 174), its CI was wide and spanned both no effect and an effect comparable to the female estimate, and neither the sex nor age interaction term approached significance. The divergence in statistical significance across strata may therefore reflect differences in power rather than genuine effect modification. The second is substantive: a real sex difference would not be unexpected, since meta-analytic evidence indicates that women report somewhat higher emotional exhaustion than men [34], and the exhaustion–depression relationship may itself be gendered. Because the interaction tests were underpowered, they cannot adjudicate between these explanations. We therefore refrain from concluding either that the differential association occurs only in women and younger workers or that it is uniform across strata. Larger samples with more work-related cases will be required to clarify whether it genuinely varies by sex or age.

### 4.5. Limitations and Generalizability

Several limitations apply. First, the cross-sectional design precludes establishing temporal order, particularly for exhaustion. Its differential association is equally compatible with work demands producing exhaustion that contributes to work-attributed depression and with work-attributed depression increasing reported exhaustion. The negative control design does not resolve this. It provides evidence against explanations that would affect both outcomes equally, including unmeasured confounding shared by the two depression groups, generic negative affectivity, and a uniform tendency to over-report health problems or work strain. It does not rule out reverse causation or bias specific to the work-attributed group, such as heightened reporting of work as draining among those who attribute their depression to work. This latter possibility cannot be excluded and is why our causal language remains cautious.

Second, the exposures and outcome are self-reported and measured contemporaneously, raising the possibility of common-method bias [35], because depressed mood could influence both symptom reporting and work-attribution. Third, and most consequentially for interpretation, the outcome was based on a single self-reported item paired with a second, independently unvalidated item asking whether the condition was caused by work. Neither item has been validated against a diagnostic interview or established symptom scale, and the KWCS provides no reference standard against which they can be calibrated. The construct-validity evidence presented here is therefore internal to the survey and does not substitute for criterion validation. Such single-item measures are susceptible to misclassification in both directions, while the attribution item asks respondents to make a causal judgement that may be influenced by compensation-seeking, social desirability, illness beliefs, or salient recent work stressors rather than the condition’s aetiology. The findings should therefore be interpreted as evidence about self-reported attribution, not clinically ascertained work-related depression. Validation of both items against diagnostic assessment is needed before measures of this kind are used to guide workplace policy.

Fourth, the two exposures were measured asymmetrically: engagement comprises three items, whereas exhaustion comprises only two, and the exhaustion items, unlike the engagement items, have not been validated in Korean workers. A shorter scale may be less reliable, potentially attenuating its association. In these data, however, this concern does not appear to materialize: the two-item exhaustion score was internally more consistent than the three-item engagement score (Spearman–Brown 0.88 versus alpha 0.80). Thus, differential attenuation is unlikely to explain why only exhaustion showed a differential association. Nevertheless, the exhaustion score has not been validated against an external criterion in this population, and the two subscales differ in the breadth of content they capture.

Fifth, residual misclassification of work- versus non-work-related depression could bias the specificity estimate in either direction.

Sixth, measurement equivalence of exhaustion across groups cannot be fully guaranteed, despite adjustment and matching. Internal consistency was near-identical across the three groups (Supplementary Table S6), which is consistent with equivalent measurement but insufficient to establish it. Relatedly, the assumed causal structure is asymmetric: exhaustion is positioned as an exposure and engagement as a descendant of depression. Although this asymmetry is substantively motivated and does not affect the estimates (Section 4.3), it shapes interpretation of the engagement result. A reader who rejects this positioning would interpret the same null finding only as evidence that engagement fails to discriminate between the two depression groups, without inferring that it partly reflects depressed mood. The present data cannot adjudicate between these interpretations.

Seventh, because the sample comprised currently employed wage workers, a healthy worker effect may have operated on both exposures [36]. For exhaustion, attrition of workers whose work-attributed exhaustion prompted sick leave or labor-market exit would attenuate rather than inflate the observed specificity contrast. For engagement, the direction is less favorable to our interpretation: if workers with the lowest engagement disproportionately left employment, particularly when their depression was work-attributed, the association could be artificially weakened, making engagement appear non-specific. However, the near-identical engagement levels in the two depression groups (6.6 and 6.5) provide little indication of this.

Eighth, although our analysis plan, including the sensitivity analyses reported in Section 3.4 and the Supplementary Material, was specified before analysis, it was not publicly registered. Readers therefore have our reported plan rather than an independently verifiable record of its prespecification.

Finally, depression was uncommon (weighted prevalence, 2.15%), and the work-related subgroup was small (*n* = 270), making high-category estimates imprecise and the stratified analyses clearly underpowered. This limitation is most pronounced in the sex-stratified estimates (Section 4.4). Lack of statistical significance within a small stratum does not establish absence of an association; accordingly, all stratified results are treated as exploratory and hypothesis-generating. The continuous specificity contrast estimated in the full sample was significant and consistent across specifications and forms the basis of our conclusions. Adjustment also controls only for the average confounding effect of the covariates; it does not indicate whether the differential association of exhaustion varies across their levels. The small number of work-related cases precluded systematic stratification beyond sex and age (Supplementary Table S3); therefore, effect modification by other characteristics cannot be excluded.

Several features nonetheless support the generalizability of these findings. The analysis covered the entire wage-earning workforce rather than a single occupation or industry, and the use of the complex-survey design with post-stratification weights produced estimates representative of Korean wage workers in 2023. The dissociation also persisted across stepwise and additional adjustments, propensity-score matching, and stratified analyses, supporting robustness to analytic choice. Most importantly, the work-/non-work-related specificity contrast directly interrogates the attribution mechanism by isolating the differential association from generic correlates of low mood. Thus, the central finding relies on an internal comparison that is less sensitive to the overall prevalence or composition of depression than a single-outcome association. These features make it plausible that the qualitative pattern—exhaustion as differentially associated and low engagement as non-specific—would extend across subgroups of the employed wage-earning population.

At the same time, the findings do not extend to the self-employed workers, employers, or unpaid family workers, who were excluded by design because work-attribution of depression lacks a clear employment referent for them. Whether exhaustion is differentially associated with work-attributed depression in these groups remains unclear. The healthy-worker survivor effect also limits generalizability because restricting the sample to currently employed workers may remove precisely those with the most extreme exposure levels. Transportability to other national or cultural contexts, where the meaning of work-attribution and the structure of employment may differ, likewise remains to be established.

### 4.6. Future Research Directions

The limitations in Section 4.5 point to a specific research agenda, grouped under three headings in descending order of importance.

First, and most pressingly, is measurement. Validation of single-item work-attribution measures against structured clinical assessment would replace an assumed misclassification rate with a known one. Cognitive interviews could also establish how workers interpret questions about causation—whether they draw on illness beliefs, the salience of recent stressors, or the prospect of compensation as much as on aetiology. Until this work is done, findings of this kind describe the behavior of an attribution rather than a diagnosis.

Second is temporal order, which no cross-sectional design can establish. The decisive test would determine whether baseline exhaustion predicts incident work-attributed depression among workers free of depression at baseline, with attribution recorded prospectively rather than reconstructed. Applying the negative-control logic longitudinally—so that exhaustion predicts incident work-attributed, but not incident non-work-attributed, depression—would provide a substantially stronger basis for interpretation. For engagement, a cross-lagged design would be more informative, since the relevant question is whether reduced engagement precedes depressive symptoms, follows them, or does both.

Third is scope and precision. Pooling multiple survey waves or comparable national surveys could address questions that a single survey cannot: whether the differential association varies by sex, age, occupation or industry; whether it extends to self-employed workers and other groups excluded here; and whether it transports to labor markets where the institutional consequences of attributing illness to work differ from those in Korea.

### 4.7. Practical Implications

If replicated in designs that establish temporal order, these associations could inform how workplace mental health data are used. We frame the implications as conditional propositions rather than recommendations because a cross-sectional study cannot support recommendations.

For surveillance, the practical distinction is between two indicators that are often collected together and treated as interchangeable summaries of workplace mental health. Exhaustion tracked the work-attribution of depression, whereas engagement did not. An organization monitoring aggregate well-being may reasonably use either, but an organization seeking to identify distress plausibly connected to working conditions, and therefore potentially modifiable by changing them, has reason to favor exhaustion-based items. Conversely, a decline in engagement should not be interpreted as evidence that work is the source of the problem because engagement declined similarly among workers who did and did not attribute their depression to work.

Our findings also relate to how worker attribution is interpreted in occupational health practice and compensation assessment: whether a worker’s own account of their depression as work-related carries information beyond the belief itself. In these data, attribution tracked a measurable, work-framed exposure in a graded fashion. This does not establish that the attribution is accurate in any individual case and should not be interpreted as doing so. However, it provides evidence against treating work-attribution as uninformative at the aggregate level.

The implications for intervention design are more limited, and deliberately so. Our estimates describe associations rather than effects of change, and the study does not indicate what would follow from reducing exhaustion. The findings instead suggest where future intervention research could focus: if exhaustion is the dimension that discriminates work-attributed distress, demand-side factors such as workload, working hours, and recovery opportunities are plausible candidates for evaluation. Engagement-oriented interventions may be better justified by their general well-being benefits than by claims that they address occupationally specific distress. These are hypotheses for intervention trials, not conclusions for implementation.

## 5. Conclusions

Among Korean wage workers, exhaustion was more strongly and dose-dependently associated with work-attributed than with non-work-attributed depression, whereas work engagement was associated with depression regardless of whether it was attributed to work. This double dissociation was consistent in direction and magnitude across adjustment models, propensity-score matching, and sensitivity analyses, although the exhaustion contrast attenuated to borderline significance under some specifications. The findings indicate that workers’ attribution of depression to work is associated with a measurable work-framed exposure, and that exhaustion, but not low engagement, distinguishes depression so attributed. For surveillance, exhaustion-based indicators may therefore be more useful than engagement-based ones for identifying depression that workers connect to their work. Low engagement, although a marker of poorer well-being, may reflect depressed mood of any origin rather than a distinctly occupational one. These cross-sectional associations derive from a single national survey, resting on self-reported and unvalidated measures of depression and its attribution. They require confirmation in longitudinal designs using validated instruments before guiding practice.

## Figures and Tables

**Table 1:** Characteristics of the complete-case analytic sample by depression status (KWCS 2023, wage workers; *N* = 25,776; survey-weighted percentages and means, design-based *p*-values).

Characteristic	No Depression (*n* = 25,235)	NWR-Depression (*n* = 271)	WR-Depression (*n* = 270)	*p*-Value
Weighted %, within sample	98.06	0.94	1.00	—
Exposures				
Exhaustion (0–8), mean (SD)	3.8 (1.7)	4.1 (1.6)	4.6 (1.6)	<0.01
Engagement (0–12), mean (SD)	7.5 (2.1)	6.5 (2.5)	6.6 (2.4)	<0.01
Demographic				
Age (years), mean (SD)	44.5 (13.7)	51.5 (14.8)	46.0 (13.7)	<0.01
Female, *n* (%)	13,337 (45.1)	200 (67.2)	167 (52.7)	<0.01
University degree or above, *n* (%)	9826 (43.6)	58 (23.5)	74 (29.2)	<0.01
Married, *n* (%)	18,657 (69.9)	217 (76.4)	199 (72.2)	0.20
Household size, mean (SD)	2.2 (0.9)	2.1 (1.0)	2.1 (0.9)	0.43
Socioeconomic				
Personal monthly income (10^4^ KRW), mean (SD)	295.9 (135.1)	238.2 (139.0)	280.4 (117.2)	<0.01
Household income contribution, *n* (%)				0.47
Primary earner	15,224 (58.6)	163 (53.4)	168 (60.4)	
Not primary	7191 (30.6)	82 (36.2)	82 (31.4)	
Equal contribution	2820 (10.8)	26 (10.3)	20 (8.2)	
Financial strain (income < expenditure), *n* (%)	5895 (22.5)	115 (40.6)	103 (36.8)	<0.01
Employment/structural				
Occupation, *n* (%)				<0.01
Non-manual	11,629 (50.5)	88 (38.1)	108 (42.9)	
Service/sales	6289 (17.4)	70 (19.2)	75 (18.0)	
Manual	7317 (32.1)	113 (42.8)	87 (39.1)	
Employment status, *n* (%)				<0.01
Permanent	20,697 (77.4)	178 (60.3)	221 (76.4)	
Temporary	3637 (18.7)	77 (34.4)	35 (17.3)	
Daily	901 (4.0)	16 (5.3)	14 (6.3)	
Weekly working hours, mean (SD)	39.0 (10.3)	33.9 (13.2)	41.4 (9.5)	<0.01
Shift type, *n* (%)				<0.01
Non-shift	23,392 (92.7)	248 (93.3)	234 (87.0)	
Shift	1115 (4.4)	17 (5.4)	24 (9.4)	
Rotating	728 (2.9)	6 (1.3)	12 (3.6)	
Workplace size, *n* (%)				0.38
1–9	11,134 (40.8)	127 (48.4)	115 (39.9)	
10–49	8805 (36.4)	90 (32.2)	94 (39.2)	
50–299	3909 (16.7)	46 (16.1)	48 (15.9)	
300+	1387 (6.0)	8 (3.3)	13 (5.0)	
Sector, *n* (%)				0.08
Private	21,581 (86.6)	216 (79.3)	225 (85.3)	
Public	2869 (10.9)	46 (17.9)	35 (12.3)	
Public–private	479 (1.7)	5 (2.2)	6 (1.8)	
NGO	306 (0.8)	4 (0.6)	4 (0.5)	
Other health indicators (not adjusted)				
WHO-5 well-being (0–100), mean (SD)	60.6 (20.0)	47.9 (23.5)	43.7 (22.0)	<0.01
Sleep problems (0–12), mean (SD)	2.0 (2.2)	4.2 (2.8)	4.4 (3.0)	<0.01
Chronic disease (6+ months), *n* (%)	1202 (4.1)	50 (17.9)	39 (13.2)	<0.01

The two rows presenting the main findings are exhaustion and engagement; the remaining rows describe the covariate set. Values are unweighted numbers and survey-weighted percentages for categorical variables, or survey-weighted mean (SD) for continuous variables; *p*-values are design-based tests across the three groups. This table describes the complete-case modeling sample (*N* = 25,776). Korean Working Conditions Survey (KWCS); standard deviation (SD); Korean won (KRW); WHO-5 Well-Being Index (WHO-5); non-work-related depression (NWR); work-related depression (WR). Sleep problems, WHO-5 well-being, and chronic disease are shown descriptively and were not adjustment variables.

**Table 2:** Per-point ORs for work-related and non-work-related depression by exhaustion and engagement (KWCS 2023, wage workers; survey-weighted, design-based 95% CIs; complete-case, *N* = 25,776).

Exposure (per 1-Point Increase)	WR vs. No-Dep	NWR vs. No-Dep	WR vs. NWR (Specificity)
Exhaustion (0–8)			
Crude	1.36 (1.22–1.51) *	1.10 (1.01–1.21) *	1.23 (1.06–1.43) *
Adjusted (M7)	1.33 (1.19–1.49) *	1.14 (1.05–1.25) *	1.17 (1.01–1.36) *
Engagement (0–12)			
Crude	0.83 (0.77–0.89) *	0.82 (0.76–0.89) *	1.01 (0.92–1.11)
Adjusted (M7)	0.86 (0.80–0.93) *	0.88 (0.82–0.95) *	0.97 (0.88–1.07)

Values are odds ratios (ORs) with 95% confidence intervals (CIs) per one-point increase. Crude = unadjusted; M7 = fully adjusted (age, sex, education, marital status, personal income, household income contribution, income–expenditure balance, shift type, sector, weekly working hours, workplace size, employment status, occupation). The specificity contrast (WR vs. NWR) restricts the sample to the two depression groups. Category-specific estimates are presented in Table 3. WR (*n* = 270); NWR (*n* = 271); no-dep, no depression (*n* = 25,235). All models use the same complete-case sample; analytic *n*: WR vs. no-dep, 25,505; NWR vs. no-dep, 25,506; specificity, 541. * CI excludes 1.0.

**Table 3:** Category-specific (dose-level) ORs for work-related and non-work-related depression by exhaustion and engagement, showing the graded specificity contrast (KWCS 2023, wage workers; survey-weighted, design-based 95% CIs; complete-case *N* = 25,776).

Exposure/ Category	WR vs. No-Dep, Crude	WR vs. No-Dep, M7	NWR vs. No-Dep, Crude	NWR vs. No-Dep, M7	WR vs. NWR (Specificity), Crude	WR vs. NWR (Specificity), M7
Exhaustion (0–8)						
0–2 (ref)	1.00	1.00	1.00	1.00	1.00	1.00
3–4	2.27 (1.35–3.81) *	2.11 (1.22–3.62) *	1.31 (0.88–1.96)	1.60 (1.09–2.34) *	1.73 (0.90–3.31)	1.31 (0.67–2.56)
5–6	3.72 (2.15–6.44) *	3.37 (1.91–5.98) *	1.45 (0.94–2.24)	1.79 (1.17–2.73) *	2.57 (1.29–5.14) *	1.84 (0.89–3.81)
7–8	6.65 (3.44–12.84) *	6.18 (3.11–12.28) *	1.57 (0.70–3.53)	1.95 (0.88–4.33)	4.23 (1.50–11.95) *	2.77 (0.97–7.90)
Engagement (0–12)						
0–3 (ref)	1.00	1.00	1.00	1.00	1.00	1.00
4–6	0.46 (0.27–0.78) *	0.52 (0.30–0.90) *	0.36 (0.22–0.57) *	0.46 (0.29–0.73) *	1.29 (0.65–2.56)	1.08 (0.54–2.16)
7–9	0.31 (0.18–0.54) *	0.38 (0.22–0.68) *	0.20 (0.12–0.33) *	0.31 (0.19–0.52) *	1.55 (0.76–3.15)	1.19 (0.57–2.49)
10–12	0.23 (0.12–0.44) *	0.31 (0.16–0.60) *	0.20 (0.11–0.39) *	0.35 (0.19–0.67) *	1.13 (0.46–2.79)	0.87 (0.35–2.17)

Values are OR (95% CI). Reference = lowest category. Crude = unadjusted; M7 = fully adjusted, as presented in Table 2. The exhaustion specificity contrast increased monotonically across categories (crude, 1.73 → 2.57 → 4.23; M7, 1.31 → 1.84 → 2.77), indicating a dose-dependent pattern. The highest M7 category estimate was borderline (7–8: OR 2.77, 95% CI, 0.97–7.90, *p* = 0.057), reflecting reduced power from categorization, whereas the continuous specification was significant (Table 2). * CI excludes 1.0.

## Data Availability

The Korean Working Conditions Survey data are publicly available from the Occupational Safety and Health Research Institute at https://oshri.kosha.or.kr.

## Supplementary Materials

The following supporting information can be downloaded at https://www.mdpi.com/article/10.3390/healthcare14183113/s1. Figure S1: assumed causal structure (directed acyclic graph); Figure S2: survey-weighted score distributions; Table S1: robustness across adjustment and sensitivity models; Table S2: effect of additional adjustment for sleep; Table S3: stratified specificity contrasts; Table S4: propensity-score-matched comparisons; Table S5: covariate balance; Table S6: internal consistency of the exposure subscales; Table S7: post hoc sensitivity analysis using a stricter case definition; STROBE checklist.

## Abbreviations

CI: confidence interval; DAG: directed acyclic graph; KRW: Korean won; KWCS: Korean Working Conditions Survey; NWR: non-work-related depression; OR: odds ratio; OSHRI: Occupational Safety and Health Research Institute; SD: standard deviation; SMD: standardized mean difference; WHO-5: WHO-5 Well-Being Index; WR: work-related depression.
