# Supplementary material for: Exhaustion, Not Low Engagement, Differentiates Work-Related and Non-Work-Related Depression in Korean Wage Workers: A Negative-Control Analysis
Source: Healthcare (Basel). 2026 Sep 21;14(18):3113. doi: 10.3390/healthcare14183113 (PMC13606196; doi:10.3390/healthcare14183113)
Supplement: Supplementary file 1 [file healthcare-14-03113-s001.zip › healthcare-4415175-supplementary.pdf]

Supplementary Materials

Exhaustion, but Not Low Engagement, Distinguishes Work-Related from Non-Work-Related Depression Among Korean Workers: A Negative Control Analysis

1. Supplementary Figures

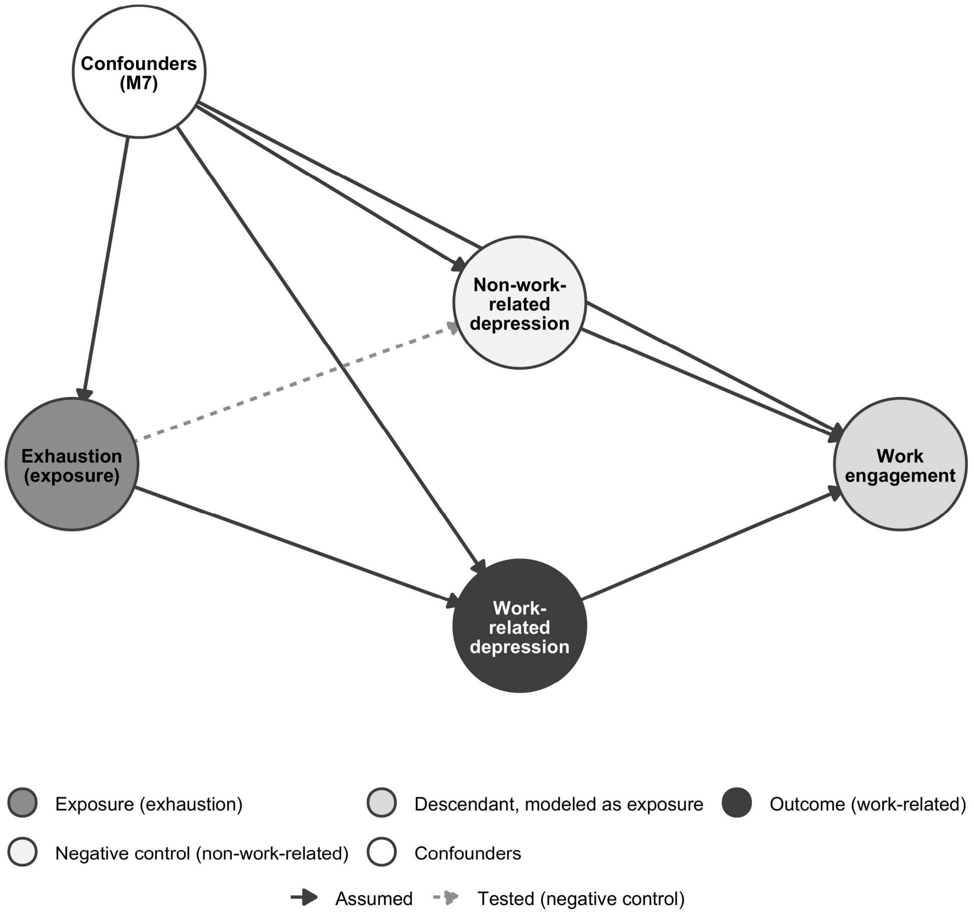

Figure S1. Assumed causal structure (directed acyclic graph) used for a priori covariate selection. Exhaustion is the exposure of interest; work-related depression is the outcome and non-work-related depression the negative control outcome. The confounder set (M7) comprises the variables listed in the Methods. Solid arrows denote assumed causal relationships; the dashed arrow denotes the path interrogated by the negative control design (whether exhaustion is associated with non-work-related depression). Work engagement is modeled as an exposure in the analysis but is depicted as a descendant of depression, on the grounds of the conceptual overlap between its components and anhedonia; it is therefore not mutually adjusted with exhaustion in the primary models. This positioning is an a priori assumption and is not established by these data: it is the assumption on which the analysis is most dependent, it cannot be verified cross-sectionally, and the mutually adjusted model reported in Table S1, which does not rely on it, yields the same pattern. Sleep problems, whose causal position relative to the exposures is ambiguous, are not included in the primary structure and are examined only in sensitivity analyses.

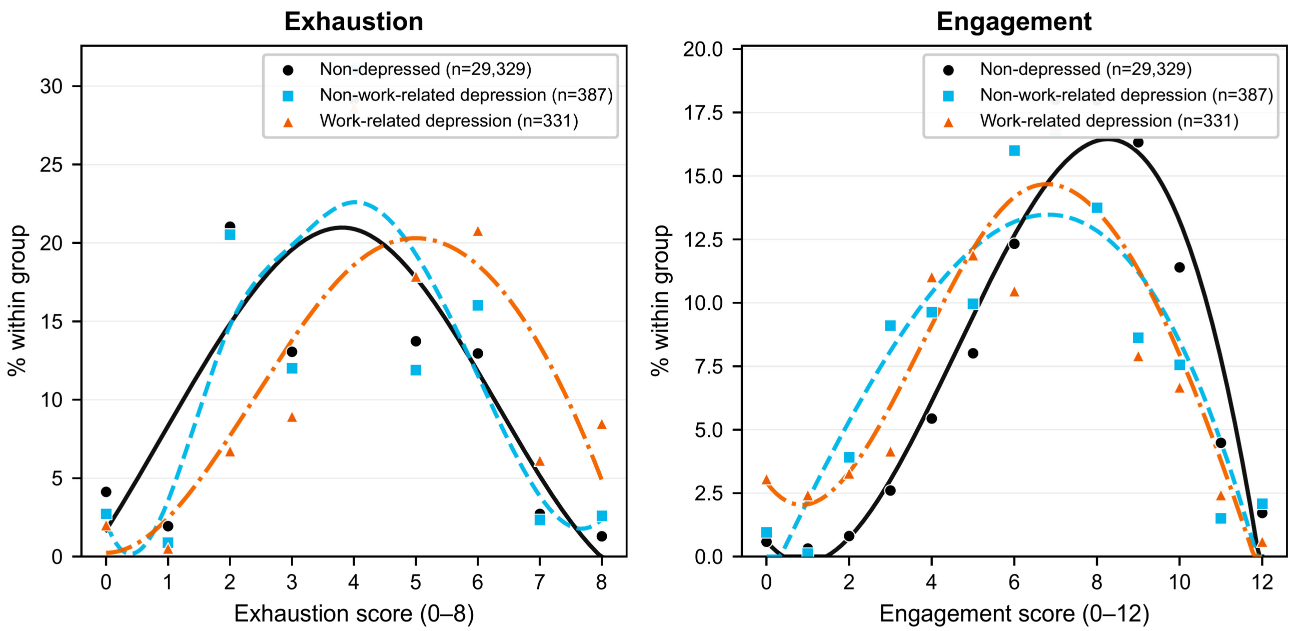

Figure S2. Survey-weighted score distributions for exhaustion (left) and work engagement (right) within each group (no depression, non-work-related depression, work-related depression), shown for the full analytic sample (n = 29,329 / 387 / 331; non-missing exposures), which is larger than the complete-case sample used for the regression models (n = 25,235 / 271 / 270). Each group sums to 100%. Markers are the weighted percentage at each score; curves are smoothing-spline fits. For exhaustion, the work-related depression group is shifted toward higher scores, whereas the non-work-related and non-depressed groups largely overlap; for engagement, both depression groups are shifted toward lower scores relative to the non-depressed.

2. Supplementary Tables

Table S1. Robustness of the per-point specificity contrast (work-related vs non-work-related depression) across stepwise adjustment and additional sensitivity models (KWCS 2023, wage workers; survey-weighted unless noted; complete-case N=25,776; specificity contrast n=541 unless noted).

Model	Exhaustion OR (95% CI)	p-value	Engagement OR (95% CI)	p-value
Stepwise adjustment				
Crude	1.23 (1.06–1.43)	<0.01	1.01 (0.92–1.11)	0.84
M1 (+ demographic)	1.18 (1.01–1.37)	0.034	1.00 (0.91–1.09)	0.94
M2 (+ income)	1.16 (1.00–1.35)	0.056	0.98 (0.90–1.07)	0.64
M3 (+ household income, balance)	1.16 (1.00–1.35)	0.053	0.97 (0.89–1.06)	0.53
M4 (+ shift type)	1.15 (0.99–1.34)	0.071	0.97 (0.89–1.07)	0.54
M5 (+ sector, working hours)	1.16 (1.00–1.35)	0.046	0.99 (0.90–1.09)	0.82
M6 (+ workplace size, employment status)	1.17 (1.01–1.36)	0.039	0.98 (0.89–1.08)	0.64
M7 (+ occupation; fully adjusted)	1.17 (1.01–1.36)	0.040	0.97 (0.88–1.07)	0.57
Additional sensitivity models (each adds to M7)				
+ Mutual adjustment (both exposures)	1.19 (1.02–1.39)	0.024	0.94 (0.86–1.04)	0.24
+ Job autonomy/control†	1.20 (1.03–1.39)	0.022	0.97 (0.88–1.07)	0.60
+ Chronic disease‡	1.18 (1.01–1.38)	0.035	0.97 (0.88–1.07)	0.51
+ Workplace verbal abuse/harassment	1.17 (1.01–1.36)	0.039	0.98 (0.89–1.08)	0.70
+ Night/weekend work§	1.16 (1.00–1.35)	0.054	0.97 (0.88–1.07)	0.58
Industry substituted for occupation	1.17 (1.01–1.35)	0.039	0.97 (0.88–1.07)	0.53
Unweighted (design-based weights removed)	1.18 (1.05–1.33)	<0.01	0.97 (0.90–1.05)	0.46

*Values are odds ratios (95% CI) per one-point increase in the exposure score, for the specificity contrast (WR vs NWR depression). The point estimate for exhaustion specificity remained between 1.15 and 1.23 across all models and was statistically significant in the crude, M1, M5, M6, and M7 models and in most additional models; it fell just short of significance at M2 and M3 (addition of personal and household income), at M4 (addition of shift type) and under adjustment for night/weekend work. Engagement specificity was non-significant in every model. Confidence limits are rounded to two decimals, so models whose lower limit prints as 1.00 may fall on either side of $p = 0.05$ (compare M2 and M5). KWCS, Korean Working Conditions Survey; OR, odds ratio; CI, confidence interval; SD, standard deviation; SMD, standardized mean difference; WR, work-related depression; NWR, non-work-related depression; no-dep, no depression; WHO-5, WHO-5 Well-Being Index. †n=530 (autonomy items missing for some respondents). ‡n=537. §n=535.

Table S2. Effect of additional adjustment for sleep problems on the per-point associations of exhaustion and engagement with depression (KWCS 2023, wage workers; survey-weighted; complete-case). This model is presented to examine the asymmetry of attenuation under adjustment for sleep, not as a preferred specification; the causal role of sleep cannot be established in cross-sectional data.

Contrast / exposure	M7 (without sleep) OR (95% CI)	p-value	M7 + sleep OR (95% CI)	p-value
WR vs no-depression				
Exhaustion	1.33 (1.19–1.49)	<0.01	1.21 (1.08–1.35)	<0.01
Engagement	0.86 (0.80–0.93)	<0.01	0.87 (0.80–0.94)	<0.01
NWR vs no-depression				
Exhaustion	1.14 (1.05–1.25)	<0.01	1.06 (0.97–1.16)	0.23
Engagement	0.88 (0.82–0.95)	<0.01	0.88 (0.82–0.95)	<0.01
WR vs NWR (specificity)				
Exhaustion	1.17 (1.01–1.36)	0.040	1.15 (0.99–1.34)	0.071
Engagement	0.97 (0.88–1.07)	0.57	0.98 (0.89–1.07)	0.63

*Values are odds ratios (95% CI) per one-point increase. The exhaustion–NWR-depression association was almost entirely accounted for by sleep (1.14 → 1.06, becoming non-significant), whereas the exhaustion–WR-depression association persisted (1.33 → 1.21, remaining significant). The component of the exhaustion association that is differential to work-attributed depression was therefore largely unaffected by adjustment for sleep, whereas the component shared with depression of any attribution was substantially attenuated. The specificity contrast itself became borderline under this adjustment (1.15; 95% CI, 0.99–1.34), as is to be expected when the comparison group's association is driven toward the null in a small subgroup; the informative feature is the asymmetry of the attenuation rather than the significance of the contrast under this admittedly overadjusted model. M7 covariates as in the main text; "M7 + sleep" adds the sleep-problem score.

Table S3. Continuous (per-point) specificity contrast (WR vs NWR depression) within strata of sex and age, and tests for effect modification (KWCS 2023, wage workers; M7-adjusted, survey-weighted; complete-case).

Stratum-specific specificity (OR per 1-point increase, WR vs NWR)

Stratum	Exhaustion OR (95% CI)	p-value	Engagement OR (95% CI)	p-value	N
Male	1.04 (0.81–1.34)	0.77	1.02 (0.84–1.22)	0.87	174
Female	1.30 (1.09–1.55)	<0.01	0.94 (0.85–1.05)	0.26	367
Age <45	1.33 (1.01–1.75)	0.046	1.03 (0.89–1.20)	0.67	171
Age ≥45	1.12 (0.92–1.37)	0.25	0.93 (0.82–1.04)	0.22	370

Tests for effect modification (interaction terms)

Exposure x modifier	Interaction OR (95% CI)	p-value
Exhaustion x sex	1.20 (0.91–1.60)	0.20
Exhaustion x age group	0.95 (0.71–1.27)	0.72
Engagement x sex	0.98 (0.82–1.17)	0.79
Engagement x age group	0.87 (0.73–1.04)	0.12

*No interaction term reached significance, and there is therefore no statistically detectable effect modification of the specificity contrast by sex or age. These stratified analyses are underpowered by construction, since the specificity contrast is estimated within the depressed subsample alone (n = 541 in total; n = 174 among men), and they are reported as exploratory. Differences between strata

in statistical significance should not be read as evidence of effect modification, and the absence of significance within a small stratum is not evidence that the association is absent there; the data cannot distinguish differing power from genuine heterogeneity.

Table S4. Propensity-score-matched paired comparisons of exhaustion and engagement across depression-group pairs (KWCS 2023, wage workers). Each pair was matched separately (1:1, nearest-neighbour on the propensity score estimated from all Table 1 covariates); covariate balance after matching was acceptable in every pair: the maximum post-matching standardized mean difference was 0.098 for the WR-vs-NWR pair, 0.144 for the NWR-vs-no-depression pair, and 0.169 for the WR-vs-no-depression pair, with the great majority of covariates below 0.10 in all three (Table S5). Exposure scores were compared with paired tests.

Matched pair (n pairs)	Exposure	Group A mean (SD)	Group B mean (SD)	Paired p
WR vs NWR (208)	Exhaustion (0–8)	WR 4.58 (1.69)	NWR 4.22 (1.61)	0.023
	Engagement (0–12)	WR 6.60 (2.49)	NWR 6.77 (2.42)	0.451
WR vs no-depression (285)	Exhaustion (0–8)	WR 4.62 (1.66)	No-dep 4.11 (1.63)	<0.01
	Engagement (0–12)	WR 6.59 (2.53)	No-dep 7.35 (2.11)	<0.01
NWR vs no-depression (287)	Exhaustion (0–8)	NWR 4.05 (1.63)	No-dep 3.83 (1.75)	0.137
	Engagement (0–12)	NWR 6.60 (2.45)	No-dep 7.06 (2.36)	0.022

*The matched comparisons reproduce the double dissociation seen in regression. For exhaustion, the work-related group differed from both the non-work-related group and the non-depressed group, whereas the non-work-related group did not differ from the non-depressed group (p = 0.137)—the pattern expected of a negative control outcome. For engagement, both depression groups were lower than the non-depressed group, but the two depression groups did not differ from each other (p = 0.451)—a non-specific pattern. A 1:1:1 triplet matching of all three groups simultaneously was also attempted but did not achieve balance (maximum post-matching SMD 0.54), because the non-work-related group differed structurally (older, lower income); separate pairwise matching was therefore used. Full pre- and post-matching covariate balance is reported in Table S5. Paired mean differences (95% CI) for the comparisons discussed in the main text were: exhaustion, work-related versus non-work-related +0.36 (0.05 to 0.67) and non-work-related versus no depression +0.22 (−0.07 to 0.50); engagement, work-related versus non-work-related −0.17 (−0.63 to 0.28) and non-work-related versus no depression −0.45 (−0.84 to −0.07). WR, work-related depression; NWR, non-work-related depression.

Table S5. Covariate balance before and after propensity-score matching, by depression-group pair (KWCS 2023, wage workers). Values are standardized mean differences (SMD). An |SMD| below 0.10 after matching is conventionally taken to indicate good balance (Austin, Stat Med 2009) and below 0.25 acceptable balance (Rubin, Health Serv Outcomes Res Methodol 2001); the observed maxima are given in the note below.

Covariate	WR vs NWR (n=208 pairs) SMD pre → post	WR vs no-dep (n=285 pairs) SMD pre → post	NWR vs no-dep (n=287 pairs) SMD pre → post
Age	−0.304 → 0.042	0.105 → 0.008	0.399 → 0.040
Household size	0.131 → −0.015	0.011 → −0.028	−0.123 → −0.071
Personal income	0.386 → −0.034	−0.069 → −0.148	−0.431 → −0.057
Weekly working hours	0.547 → −0.020	0.185 → −0.114	−0.380 → −0.058
Female	−0.260 → 0.021	0.162 → −0.029	0.427 → 0.008

University degree+	0.134 → 0.033	−0.229 → −0.008	−0.365 → 0.000
Married	−0.128 → 0.056	−0.006 → −0.040	0.121 → −0.017
Financial strain	−0.094 → −0.030	0.311 → 0.022	0.408 → −0.091
Income contribution: primary	0.037 → 0.059	0.038 → 0.043	0.001 → 0.000
Income contribution: not primary	0.005 → −0.062	0.031 → −0.030	0.027 → −0.023
Income contribution: equal	−0.060 → 0.017	−0.117 → −0.038	−0.057 → 0.012
Occupation: non-manual	0.129 → 0.030	−0.127 → −0.050	−0.257 → 0.030
Occupation: service/sales	0.068 → −0.098	0.074 → 0.016	0.006 → −0.040
Occupation: manual	−0.190 → 0.061	0.065 → 0.038	0.256 → 0.007
Employment: permanent	0.374 → 0.034	0.009 → −0.037	−0.364 → −0.029
Employment: temporary	−0.381 → −0.050	−0.052 → 0.053	0.329 → 0.087
Employment: daily	−0.043 → 0.021	0.071 → −0.015	0.114 → −0.093
Shift: non-shift	−0.156 → 0.032	−0.202 → −0.166	−0.046 → 0.047
Shift: shift	0.133 → −0.020	0.201 → 0.162	0.069 → −0.107
Shift: rotating	0.078 → −0.053	0.070 → 0.056	−0.008 → 0.070
Size: 1–9	−0.072 → 0.019	−0.028 → 0.043	0.044 → −0.014
Size: 10–49	0.027 → −0.021	−0.014 → −0.015	−0.041 → 0.000
Size: 50–299	0.022 → 0.000	0.053 → −0.082	0.031 → 0.010
Size: 300+	0.111 → −0.083	−0.018 → 0.000	−0.128 → 0.000
Sector: private	0.103 → 0.041	−0.037 → −0.087	−0.140 → −0.144
Sector: public	−0.114 → 0.000	0.037 → 0.053	0.150 → 0.137
Sector: public–private	0.001 → −0.081	0.009 → 0.085	0.008 → 0.084
Sector: NGO	0.001 → 0.000	0.012 → 0.169	0.011 → 0.000

*Matching and these balance statistics are unweighted. Across the 28 covariates examined, the maximum post-matching |SMD| was 0.098 for the WR-vs-NWR pair (no covariate above 0.10), 0.144 for the NWR-vs-no-depression pair (three above 0.10, the largest being sector: private), and 0.169 for the WR-vs-no-depression pair (five above 0.10, the largest being sector: NGO). Balance is therefore good for the pair on which the specificity contrast rests and acceptable for the other two; the few small residual imbalances (e.g., sector categories with sparse cells) do not affect the exposure comparisons (exposure results are reported in Table S4). WR, work-related; NWR, non-work-related; no-dep, no depression.

Table S6. Internal consistency of the two exposure subscales, overall and by depression group (KWCS 2023, wage workers; complete-case N = 25,776). Coefficients are unweighted, with survey-weighted values in parentheses.

Sample (n)	Engagement, 3 items: Cronbach's alpha	Exhaustion, 2 items: inter-item r	Exhaustion, 2 items: Spearman–Brown
Overall (25,776)	0.802 (0.785)	0.777 (0.758)	0.875 (0.862)
No depression (25,235)	0.801 (0.784)	0.777 (0.757)	0.874 (0.862)
NWR-depression (271)	0.816 (0.823)	0.788 (0.765)	0.881 (0.867)
WR-depression (270)	0.807 (0.811)	0.751 (0.768)	0.858 (0.869)

*Cronbach's alpha is reported for the three-item engagement subscale. For a two-item scale alpha is uninformative, so the inter-item correlation and the Spearman–Brown prophecy coefficient are given instead; the latter is the appropriate internal-consistency estimate for a two-item instrument and is directly comparable with alpha. The two-item exhaustion subscale was internally more consistent than the three-item engagement subscale, so the asymmetry in item count does not correspond to an asymmetry in reliability. Coefficients were near-identical across the three depression

groups, which is consistent with equivalent measurement across groups without being sufficient to establish it. WR, work-related depression; NWR, non-work-related depression.

Table S7. Post hoc sensitivity analysis using a stricter case definition (KWCS 2023, wage workers; survey-weighted; complete-case; all models M7-adjusted). Cases were restricted to respondents who both reported past-year depression and scored 50 or below on the WHO-5 Well-Being Index; the work attribution was taken from the original attribution item, so the work-related versus non-work-related structure is unchanged. Values are odds ratios (95% CI) per one-point increase in the exposure score.

Contrast / exposure	Original definition	Stricter definition (WHO-5 ≤ 50)
Exhaustion: WR vs NWR (specificity contrast)	1.17 (1.01–1.36), $p = 0.040$; n = 541	1.26 (1.04–1.52), $p = 0.020$; n = 314
Exhaustion: WR vs no depression	1.33 (1.19–1.49), $p < 0.01$	1.30 (1.11–1.51), $p < 0.01$
Exhaustion: NWR vs no depression (negative control)	1.14 (1.05–1.25), $p < 0.01$	1.07 (0.95–1.19), $p = 0.26$
Engagement: WR vs NWR (specificity contrast)	0.97 (0.88–1.07), $p = 0.57$	0.99 (0.86–1.13), $p = 0.83$
Engagement: WR vs no depression	0.86 (0.80–0.93), $p < 0.01$	0.71 (0.65–0.77), $p < 0.01$
Engagement: NWR vs no depression	0.88 (0.82–0.95), $p < 0.01$	0.76 (0.70–0.82), $p < 0.01$

*This analysis was conducted post hoc rather than as one of the pre-specified sensitivity analyses. Under the stricter definition the specificity contrast for exhaustion is not weakened but slightly strengthened, and the association with the negative control outcome, significant under the original definition, is abolished; engagement remains non-specific. Two cautions apply. Case numbers fall by roughly 40% (work-related 270 to 165; non-work-related 271 to 149), so the estimates are less precise. More importantly, WHO-5 well-being is itself a consequence of depression and is correlated with exhaustion, so conditioning on it is conditioning on a descendant of the outcome and risks collider bias; the expected direction of such bias is attenuation of the contrast rather than inflation, and the analysis is therefore best read as showing that the differential association survives a conservative restriction rather than as an improved estimate of its magnitude. WR, work-related depression; NWR, non-work-related depression.
